# Synthesis and Applications of Optical Materials

**DOI:** 10.3390/nano13020297

**Published:** 2023-01-11

**Authors:** Seung-Min Park, Bong-Hyun Jun

**Affiliations:** 1 Department of Urology, Department of Radiology, Molecular Imaging Program at Stanford, Stanford University School of Medicine, Stanford, CA 94305, USA; 2 Department of Bioscience and Biotechnology, Konkuk University, Seoul 143-701, Republic of Korea

As optical materials have shown outstanding physical and chemical characteristics in the bio, medical, electronics, energy and related fields of studies, the potential benefits of using these materials have been widely recognized [1,2]. Thus, research on many applications has been conducted using many optical materials of various shapes and compositions. This Special Issue aims to provide a range of original contributions detailing the synthesis and application of optical materials.

Our Special Issue spans optical materials that exhibit a variety of unique characteristics, including plasmonic nanomaterials, quantum dots, and carbon materials. It also includes the applications that use optical properties. This Special Issue provides eleven outstanding papers in the development of synthesis and application of optical materials.

Quantum dots (QDs) have attracted a great deal of attention in a wide range of fields related to electronics, optics, biosystems, and materials synthesis thanks to various properties distinct from conventional fluorescent dyes [3,4,5,6]. Development of highly luminescent QDs is very helpful in efficient biological use [7]. Chang et al. reported improved characteristics of CdSe/CdS/ZnS core-shell quantum dots [8]. CdSe/CdS with ZnS/ZnO shell QDs are synthesized by the one-pot method with various oleylamine (OLA) contents. QDs with a high OLA concentration exhibit diffraction peaks of ZnS/ZnO and the thermal stability of QDs with ZnS/ZnO shells exhibits better performance than those with ZnS shells. Moreover, the photoluminescence intensity of QDs with ZnS/ZnO shells shows a relatively slow decay of 7.1% compared with ZnS shells at a 85 °C/85% relative humidity aging test for 500 h.

Since a sensitive biomolecule detection system has potential uses for early detection and diagnosis of various diseases, QDs assembled on silica particles (QD^2^) have been reported for such applications [9]. Exosomes are attracting attention as new biomarkers for monitoring the diagnosis and prognosis of certain diseases. Kim et al. reported the QD^2^-based lateral flow assay for highly sensitive exosome detection [10]. Anti-CD63 antibodies were introduced on the surface of the highly bright QD^2^, and a lateral flow immunoassay with QD^2^ was conducted to detect human foreskin fibroblast (HFF) exosomes. The exosome samples embraced a wide range of concentrations from 100 to 1000 exosomes/µL, and the detection limit of their newly designed system was 117.94 exosome/μL, which was 11 times lower than the previously reported limits. Bock et al. reported lateral flow immunoassay with QD^2^ for prostate-specific antigen (PSA), which is one of the best-known biomarkers for early diagnosis of prostate cancer [11]. In particular, only a simple detection system including a smartphone and a computer software program was employed for signal transduction, because the developed system had high sensitivity by using very bright nanoprobes. The limit of PSA detection was 0.138 ng/mL and the area under the curve was 0.852. The system did not show any false-negative results while 47 human serum samples were analyzed, which may have a great clinical utility in in vitro diagnostics [12].

Rapid containment of viral infectious diseases has become a major concern for global health. The QD^2^ have been employed to detect an H1N1 virus [13]. Seder et al. reported a movable layer device for rapid detection of influenza, an H1N1 virus, using QD^2^ and magnetic beads [14], a microfluidic platform that performs an immunoassay of viral antigens in a simple, automated, yet highly sensitive manner. The automated device achieves a highly sensitive magnetic bead-based sandwich immunoassay for the influenza A H1N1 virus within 32.5 min. The detection limit of the method is 5.1 × 10^−4^ hemagglutination units, which is 2 × 10^3^ times more sensitive than that of the conventional hemagglutination method and is comparable to PCR.

Carbon materials have been a great candidate as optical materials [15,16,17,18]. Limosani et al. reported successfully synthesized N-doped carbon quantum dots (N-CQDs) using a hydroxyl radical opening of fullerene with hydrogen peroxide [19]. The N-CQDs were probed for metal ion detection in aqueous solutions and during bioimaging and displayed a Cr^3+^ and Cu^2+^ selectivity shift at a higher degree of -NH_2_ functionalization, as well as HEK-293 cell nuclei marking. W.-H. Park reviewed the various characterization methods of chemical vapor deposition of monolayer graphene electrodes (CVD-MG), which are devised and developed for achieving a largescale, highly flexible, and transparent electrode [20].

The optical properties of metal nanoparticles have long been of interest in material science and applications [21,22,23,24]. The development of high efficiency dye-sensitized solar cells (DSSCs) has received tremendous attention [25,26,27]. Lee et al. studied an effect of Au NPs and scattering layer in dye-sensitized solar cells (DSSCs). Based on freestanding TiO_2_ nanotube arrays [28], they introduced Au nanoparticles (Au NPs) and a scattering layer to change the power conversion efficiency (PCE) of DSSCs. The Au NPs layer could act as a better source of electron generation because the plasmonic absorption band of Au NPs is 530 nm, which matches the dye absorbance, and a scattering layer had better light harvesting by scattering.

Surface-enhanced Raman spectroscopy (SERS) has become an essential analytical tool for various target molecules detection [29,30,31,32,33,34,35,36]. However, the direct detection of H_2_O_2_ by SERS is not possible because of its low Raman cross-section. Pham et al. reported nonenzymatic hydrogen peroxide detection using SiO_2_@Ag@Au alloy SERS NPs [37]. The peroxidase-mimicking activity of SiO_2_@Au@Ag alloy NPs in the presence of TMB was investigated using SERS for detecting H_2_O_2_. Briefly, in the presence of H_2_O_2_, the SiO_2_@Au@Ag alloy catalyzed the conversion of TMB to oxidized TMB, which was absorbed onto the surface of the SiO_2_@Au@Ag alloy. The evaluation of the SERS band to determine the H_2_O_2_ level utilized the SERS intensity of oxidized TMB bands.

The reproducible, reliable fabrication for large area SERS substrates in a low-cost remains a challenge. Luo et al. reported large area patterning of highly reproducible and sensitive SERS sensors based on 10 nm annular gap arrays using a patterning method based on nanosphere lithography and adhesion lithography technics [38].

Various composites and shapes of optical materials are introduced [39,40]. The high optical absorption and emission of bidimensional MoS_2_ are fundamental properties for optoelectronic and biodetection applications. Cortijo-Campos et al. reported size effects in single- and few-layer MoS_2_ nanoflakes on Raman phonons and photoluminescence [41]. Hossain et al. reported recent studies of NIR (near infrared)-light responsive materials for photothermal cell treatments [42]. In their review, various nanomaterials such as metal and carbon-based nanomaterials are compared systematically.
